# Micro RNAs as potential biomarkers in tuberculosis: A systematic review

**DOI:** 10.1016/j.ncrna.2021.12.005

**Published:** 2022-01-01

**Authors:** Bijay Pattnaik, Niharika Patnaik, Saurabh Mittal, Anant Mohan, Anurag Agrawal, Randeep Guleria, Karan Madan

**Affiliations:** a Department of Pulmonary, Critical Care and Sleep Medicine, All India Institute of Medical Sciences (AIIMS), New Delhi, India; b Centre of Excellence in Asthma & Lung Disease, Molecular Immunogenetics Lab, CSIR-Institute of Genomics & Integrative Biology, New Delhi, India

**Keywords:** microRNAs, Profiling, Diagnostic biomarkers, Tuberculosis

## Abstract

Tuberculosis (TB) remains a major infectious disease across the globe. With increasing TB infections and a rise in multi-drug resistance, rapid diagnostic modalities are required to achieve TB control. Radiological investigations and microbiological tests (microscopic examination, cartridge-based nucleic acid amplification tests, and cultures) are most commonly used to diagnose TB. Histopathological/cytopathological examinations are also required for an accurate diagnosis in many patients.

The causative agent, *Mycobacterium tuberculosis* (Mtb), is known to circumvent the host's immune system. Circulating microRNAs (miRNAs) play a crucial role in biological pathways and can be used as a potential biomarker to detect tuberculosis. miRNAs are small non-coding RNAs and negatively regulate gene expression during post-transcriptional regulation. The differential expression of miRNAs in multiple clinical samples in tuberculosis patients may be helpful as potential disease biomarkers. This review summarizes the literature on miRNAs in various clinical samples as biomarkers for TB diagnosis.

## Abbreviation list

Mtb: Mycobacterium tuberculosis
PCR: Polymerase chain reaction
miRNA: micro- Ribonucleic Acid
CT: Computed Tomography
IFN-γ: Interferon-gamma
SIRT-1: NAD-dependent deacetylase sirtuin-1
BAL: Bronchoalveolar lavage
EBB: Endobronchial biopsy
TBLB: Transbronchial lung biopsy
EBUS-TBNA: Endobronchial ultrasound guided transbronchial needle aspiration
EBC: Exhaled breath condensate
PBMC: peripheral blood mononuclear cell
NFAT5: nuclear factor of activated T-cells 5
NFkB: Nuclear factor k beta
CXCL2: C-X-C Motif Chemokine Ligand 2
CCL3: C-C Motif Chemokine Ligand 3
IL: Interleukin
TGF-β: Transforming growth factor-beta
TLR/MyD88: toll-like receptor/myeloid differentiation factor 88
TLR: Toll like receptor
NK cell: Natural killer cell
IGF: Insulin growth factor
TNF: Tumour necrosis factor
TRAF 3: TNF receptor-associated factor 3
LPS: Lipopolysaccharide.

## 1 Introduction

Due to tuberculosis, more than 1 million people die every year in low-income and middle-income countries [1]. According to current reports, one-third of the world population is latently infected with tuberculosis [2]. However, only 5–10% of infected people develop active tuberculosis in their lifetime [3]. The causative bacillus, i.e. *Mycobacterium tuberculosis* (Mtb), commonly infects the respiratory system by inhalation and reaches the alveolar space [4]. Further, mycobacteria undergo phagocytosis by the alveolar macrophages and attribute to granuloma formation. Another report suggests that Mtb can modulate cellular processes like cytokine production and phagolysosome maturation in macrophages and dendritic cells [5]. Understanding the pathogenesis is crucial to the success of tuberculosis control programs.

For TB control, accurate and rapid modalities for the initial diagnosis are required. However, the standard methods to detect tuberculosis involve the growth of microorganisms in a selective medium that requires 3–12 weeks [6]. Smear examinations are also used to detect tuberculosis, but sputum smears have low sensitivity [7]. Although the PCR and immunological tests based evaluation of tuberculosis are rapid diagnostic methods [8], false-positive and negative results make them unreliable. Other reliable diagnosis modalities include demonstrating granulomatous inflammation in the affected organs and a compatible clinico-radiological profile [9]. The various modalities for tissue sampling in intrathoracic TB include endobronchial ultrasound-guided transbronchial needle aspiration (EBUS-TBNA), bronchoalveolar lavage (BAL), endobronchial biopsy (EBB), transbronchial lung biopsy (TBLB), or percutaneous sampling modalities [10]. These are invasive diagnostic modalities that require super-speciality facilities and expertise. To improve non-invasive and early diagnosis, the global scientific community continuously researches new diagnostic modalities.

Accumulating evidence suggests that most of the cell-mediated immune responses are controlled by microRNAs (miRNAs), and the modulation of miRNA expression associated with these biological processes is one of the crucial strategies to prevent the spread of infection [[11], [12], [13], [14], [15], [16]]. MiRNAs are small, single-stranded, non-coding RNAs that bind to the specific gene to modulate its expression [17,18]. These involve multiple cellular processes, such as cell cycle control, apoptosis, and several developmental and physiological processes [19]. Alteration of miRNAs expressions and gene expressions is associated with multiple respiratory diseases' pathogenesis.

Several studies have suggested the role of circulating miRNAs in patients with pulmonary tuberculosis and identified miRNA signatures that could discriminate between patients with active tuberculosis and healthy controls or latent tuberculosis [20]. Currently, global TB research has focused on the importance of miRNAs in the pathogenesis of tuberculosis. Several researchers have explored the role of thousands of miRNA effects in pathogenesis and attempted to identify new biomarkers for diagnosing tuberculosis in different patients' samples [[20], [21], [22]]. Given the importance of miRNAs as potential biomarkers for detecting tuberculosis, we have reviewed the literature on identifying miRNAs in different sample types and the role of the identified miRNAs in Mtb infected patients.

This review article summarizes recently discovered miRNAs isolated from different clinical samples in patients with tuberculosis. We have also highlighted their role in the pathogenesis of tuberculosis and their potential as TB biomarkers. This review may help understand how these small molecules play a crucial role in TB pathogenesis and immune response and using them as potential biomarkers to detect tuberculosis.

## 2 Methods

### 2.1 Inclusion criteria

Both PubMed and EMBASE electronic databases were screened for relevant studies up to November 2021. Abstracts and full texts were reviewed for information regarding eligibility criteria, excluding abstract-only studies and studies not in English.

### 2.2 Search strategy

A systematic search of the PubMed and EMBASE databases was performed to extract relevant studies using the following search terms: [(micro RNA OR Mirna OR snRNA) AND (tuberculosis OR tb OR tuberculous OR tubercular)]. The “Zotero” reference manager was used to create a database, and the electronic searches were added to the database. The full text of the qualifying articles was retrieved and studied in detail. The following information was extracted: (a) publication details (authors, year of publication), (b) miRNAs in inflammation and pathogenesis, and (c) miRNAs identified in different clinical samples.

### 2.3 Method of the review

Two reviewers independently screened citations and abstracts to identify articles potentially meeting the inclusion criteria. Full-text versions were retrieved and individually screened by two reviewers to determine whether they met the inclusion criteria. Disagreements in inclusion criteria were resolved through discussion with a third reviewer.

## 3 Results

**Fig. 1:**
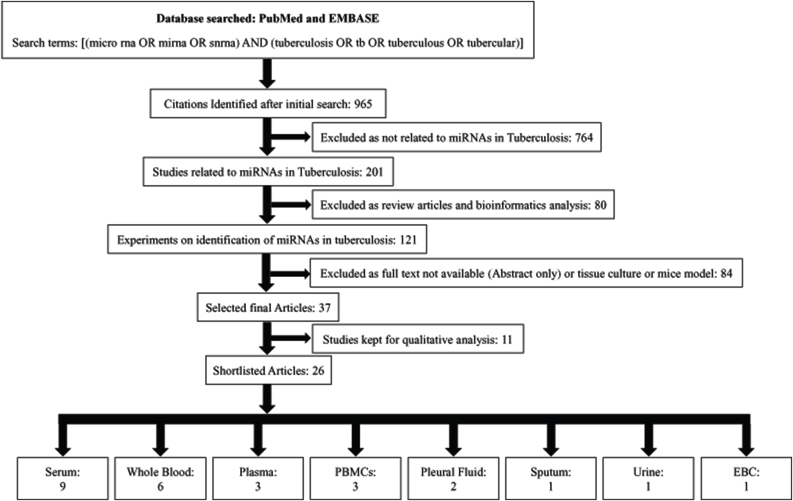
Methodology of a systematic review of the literature regarding miRNAs in Tuberculosis.

The initial search revealed a total of 965 studies, of which 764 were excluded as those studies were not related to miRNAs in tuberculosis (Fig. 1). We excluded 80 other articles by screening titles and abstracts as they were review articles and bioinformatics analysis. Further, 84 articles were excluded because of the unavailability of full text (abstract only), were cell culture studies, or mice models of tuberculosis infections. Thirty-seven studies were included in the qualitative analysis as these studies described the differential expression of miRNAs in various clinical samples of tuberculosis patients. We further shortlisted 26 articles focusing on miRNAs profiling and divided them into eight sub-groups based on the clinical samples used for miRNAs study in tuberculosis patients. A flow diagram of study screening and selection is shown in Fig. 1.

### 3.1 Identification of miRNAs in different clinical samples

Screening methods for tuberculosis based on disease biomarkers may help detect subjects in the early stages of the disease. In the last few decades, several studies have been published to identify miRNAs in tuberculosis clinical samples like whole blood, plasma, serum, peripheral blood mononuclear cells (PBMCs), sputum, Pleural fluid, Urine, and Exhaled Breath Condensates (EBCs) collected from patients with active pulmonary tuberculosis. These studies employed unbiased methods like miRNAs microarray analysis or next-generation sequencing techniques or selecting inflammatory and apoptosis-associated candidate miRNAs profiling. The RNA isolation method is crucial in all these techniques, influencing the results. Accumulating evidence suggests that miRNAs are abundantly present in exosomes. Hence, few studies have focused on exosome-vesicles associated with miRNAs isolated from any clinical samples. The identification of miRNAs from clinical samples in active tuberculosis are summarized in Table 1.

**Table 1:** Identified miRNAs in clinical samples by profiling and their roles in pathology.

S. No	Sample Type	Technique Used	microRNAs	Result	Function of miRNAs	Year	References
1	Serum	miRNAs Profiling Assay	miR-361-5p, miR-889, miR-576-3p	miR-361-5p, miR-889, miR-576-3p were up regulated in tuberculosis.	miR-361-5p targets SP-1 transcription factor, a key signaling pathway for IL-10. miR-889 are associated with respiratory system development. miR-576-3p involved in immune system development.	2012	[82]
		miRCURY LNA array	miR-3125, miR-93∗, and miR-29a	miR-3125 was found to be down regulated miR-93∗, and miR-29a were up regulated	miR-93∗ involves in promoting tumor growth by targeting the tumor suppressor gene Fus1 and integrin- 8. miR-29a is a regulator of Wnt signaling pathway genes and lipid metabolism.	2011	[83]
		TaqMan miRNA array	miR-1249, miR-1178, miR-668, miR-220b, let-7i∗, miR-941, miR-212, miR-28-3p, miR-620, miR-532-5p, miR-17, miR-151-5p, miR-20b, miR-369-3p, miR-573, miR-1263, miR103, miR-182, miR-196b, miR-130b∗, miR-1284, let-7b	miR-1249, miR-1178, miR-668 were up regulated. miR-220b, let-7i∗, miR-941, miR-212, miR-28-3p, miR-620, miR-532-5p, miR-17, miR-151-5p, miR-20b, miR-369-3p, miR-573, miR-1263, miR-103, miR-182, miR-196b, miR-130b∗, miR-1284, let-7b were down regulated.	miR-1249, let-7i, miR-28-3p, miR-103, involve in glucose metabolism. miR-1178, miR-668, miR-220b, miR-941, miR-151-5p, miR-573, miR-1263, let-7b play role in inflammation. miR-212 regulates the expression of SIRT-1 gene expression. miR-620, miR-532-5p, miR-17, miR-20b, miR-369-3p, miR-182, miR-196b, miR-130b, miR-1284 regulate cancer cell proliferation.	2015	[84]
		Solexa Sequencing	miR-17-5p, miR-20b-5p, and miR-423-5p	miR-17-5p, miR-20b-5p, and miR-423-5p were up-regulated	miR-17-5p and miR-423-5p regulate cell proliferation. miR-20b-5p regulates wound healing process.	2019	[58]
		Solexa Sequencing	miR-196b, miR-516b, miR-486-5p and miR-376c	miR-196b, miR-516b, miR-486-5p and miR-376c up regulated	miR-196b inhibits proliferation and induces apoptosis. miR-516b, miR-486-5p and miR-376c target nuclear factor of activated T-cells 5 and regulate inflammation.	2014	[43]
		TaqMan Low Density Array (TLDA)	let-7e, miR146a, miR-148a, miR-16, miR-192, miR-193a-5p, miR-25, miR-365, miR-451, miR-532-5p, miR-590-5p, miR-660, miR-885-5p, miR-223∗, miR-30e	Differentially expressed in serum samples	Let7e, miR-146a, miR-16, miR-223 modulates inflammation. miR-148a regulates B cell tolerance and autoimmunity. miR-192, miR193a-5p, miR-25, miR-365, miR-451, miR-532-5p, miR-590-5p, miR-660, miR-885-5p, miR-30e regulate cancer cell proliferation and invasion.	2013	[20]
		Solexa Sequencing	miR-21-5p, miR-92a-3p, and miR-148b-3p, miR-16	miR-21-5p, miR-92a-3p, and miR-148b-3p, miR-16 were up regulated in tuberculosis	miR-21-5p, miR-148b-3p regulates cancer cell proliferation. miR-92a-3p regulates p38 MAPK/NFκB pathway. miR-16 plays role as an anti-inflammatory miRNA.	2017	[33]
		Solexa Sequencing	miR-378, miR-483-5p, miR-22, miR-29c, miR-320b and miR-101	miR-378, miR-483-5p, miR-22, miR-29c were up regulated and miR-320b and miR-101 were down regulated in tuberculosis	miR-378, miR-483-5p, miR-22, miR-29c, miR-320b, miR-101 regulates cancer cell proliferation.	2013	[51]
		Small RNA transcriptome profiles	let-7e-5p, let-7d-5p, miR-450a-5p, miR-140-5p, miR-1246, miR-2110, miR-370-3p, miR-28-3p, and miR-193b-5p	let-7e-5p, let-7d-5p, miR-450a-5p, miR-140-5p were up regulated in latent tuberculosis infection and miR-1246, miR-2110, miR-370-3p, miR-28-3p, and miR-193b-5p were up regulated in active tuberculosis infection.	Let7e-5p plays role in immune response in TB infection. miR-450a-5p inhibits autophagy, miR-140-5p regulates T cell differentiation. miR-1246, miR-2110 regulate cancer cell proliferation. miR-370-3p targets KDR/AKT signalling pathway gene and inhibits vascular smooth muscle cell proliferation. miR-28-3p regulates B cell lymphomas. miR-193b-3p regulates TGF-β gene.	2019	[40]
2	Whole Blood	Micro array	hsa-miR-150, hsa-miR-21, hsa-miR-29c and hsa-miR-194	miR-150 was down regulated and miR-21, miR-29c, miR-194 were up regulated in tuberculosis	miR-150 negatively regulates natural killer cells. miR-21 promotes fibrosis. miR-29c targets IFN-γ and regulates immune cells. miR-194 inhibits innate antiviral immunity.	2015	[21]
		Micro array	miR-1, miR-155, miR-31, miR-146a, miR-10a, miR-125b, miR-150 and miR-29	miR-1, miR-155, miR-31, miR-146a, miR-10a, miR-125b, miR-150 were down regulated and miR-29 was up regulated	miR-1 regulate cancer cell proliferation. miR-155 regulates the adaptive immune response. miR-31 regulates lipid metabolism. miR-146a regulates IL-6 production and inflammation. miR-10 regulates TGF-β signaling pathway genes. miR-125b regulates EMT. miR-150 negatively regulates natural killer cells. miR-29c targets IFN-γ and regulates immune cells.	2016	[31]
		Micro array	miR-182, miR-355, miR-15b∗, miR-340, and miR-144	miR-182, miR-355, miR-15b∗, miR-340 were down regulated and miR-144 up regulated	miR-182, miR-355, miR-15b, miR-340, miR-144 regulate cancer cell proliferation.	2012	[85]
		TaqMan miRNAs assay	miR-26a, miR-29a, and miR-142- 3p	miR-26a, miR-29a, and miR-142- 3p were down regulated	miR-26a regulates cancer cell proliferation. miR-29a is a regulator of Wnt signaling pathway genes and lipid metabolism. miR-142-3p regulates mesenchymal cells differentiation and proliferation.	2013	[50]
		Small RNA sequencing	miR-1197, miR-1268b, miR-1275, miR-1299, miR-146a-3p, miR-150-3p, miR-150-5p, miR-30a-3p, miR-3135a, miR-342-3p, miR-3679-5p, miR-4467, miR-449a, miR-451b, miR-493-3p, miR-503-5p, miR-618, miR-629-5p, miR-6513-5p, miR-6734-3p, miR-6774-3p, miR-6802-3p, miR-708-3p, miR-708-5p, miR-874-3p, miR-874-5p, miR-876-3p, miR-877-3p, miR-92a-1-5p, miR-941	miR-1197, miR-1299, miR-30a-3p, miR-3135a, miR-4467, miR-449a, miR-451b, miR-493-3p, miR-503-5p, miR-629-5p, miR-6513-5p, and miR-941 were up regulated. miR-1268b, miR-1275, miR-146a-3p, miR-150-3p, miR-150-5p, miR-342-3p, miR-3679-5p, miR-618, miR-6734-3p, miR-6774-3p, miR-6802-3p, miR-708-3p, miR-708-5p, miR-874-3p, miR-874-5p, miR-876-3p, miR-877-3p, miR-92a-1-5p were down regulated	miR-1197, miR-1268b, miR-1299, miR-30a-3p, miR-342-3p, miR-449a, miR-451b, miR-493-3p, miR503-5p, miR-629-5p, miR-6734, miR-708, miR-874, miR-876, miR-92a-5p regulate cancer cell proliferation. miR-1275 regulates adipogenesis. miR-146a regulates IL-6 production and inflammation. miR-150 negatively regulates natural killer cells. miR-618 can regulate TIMP-1 expression which regulates MMP9 expression. miR-877-3p targets SMAD7 and regulates myofibroblast differentiation.	2018	[51]
		GeneChip miRNAs array	miR-20a, miR-20b, miR-26a, miR-106a, miR-191, miR-486, miR-3128, miR-1468, miR-3201, miR-8084	miR-20a, miR-20b, miR-26a, miR-106a, miR-191, miR-486 were up regulated and miR-3128, miR-1468, miR-3201, miR-8084 were down regulated	miR-20 helps in the cardiac development. miR-26a helps the cellular development, growth, apoptosis and metastasis. miR-106a, miR-191, miR-486, miR-1468 regulate cancer cell proliferation.	2019	[44]
3	Plasma	miRNAs micro array	let-7b-5p, let-7d-5p, let-7g-5p, miR-30b-5p, and miR-331- 3p	let-7b-5p, let-7d-5p, let-7g-5p, miR-30b-5p, and miR-331- 3p were up regulated in active tuberculosis	Let-7 miRNAs family involves in many inflammatory pathways by targeting various genes. miR-30b-3p, miR-331-3p regulates cancer cell proliferation.	2016	[41]
		Real time PCR array	miR-21-5p, miR-99b-5p, miR-29a-5p, miR-223-5p, miR-221-3p, miR-146a-5p, miR-26a-5p, miR-28-5p, miR-133a, and miR-652-3p	miR-99b-5p, miR-29a-5p, miR-223-5p, miR-221-3p and miR-28-5p were up regulated and miR-21-5p, miR-146a-5p, miR-26a-5p, miR-133a, and miR-652- 3p were down regulated in tuberculosis	miR-21 targets TGB-β signaling pathway genes and regulates fibrosis. miR-99b-5p, miR-29a-5p, miR-223-5p, miR-221-3p, miR-146a-5p, miR-28-5p, regulate cancer cell proliferation. miR-26a-5p targets ULK1 and regulates cardiac fibroblast collagen expression. miR-133a plays role in cardiac remodeling. miR-652-3p regulates endothelial cell proliferation.	2018	[47]
		Solexa Sequencing	miR-769-5p, miR-320a, miR-22-3p	miR-769-5p, miR-320a, miR-22-3p were down regulated	miR-769-5p involves in the prognosis of pancreatic cancer and non-small cell lung cancer miR-320a involves in the modulation of cytokine production, cell proliferation, migration and invasion. miR-22-3p involves in the DNA damage machinery.	2017	[86]
4	PBMCs	miRNA micro array	miR-200c, miR-193a-3p, miR-595, miR-432, miR-155, miR-9, miR-141, miR-32, miR-29b, miR-152, miR-144	miR-200c, miR-193a-3p, miR-595, miR-432, miR-155 and miR-9 were up regulated and miR-141, miR-32, miR-29b, miR-152, miR-144 were down regulated	miR-200c, miR-193a-3p, miR-432, miR-141, miR-152 regulate cancer cell proliferation. miR-595 targets SOX7 and promotes glioblastoma cell proliferation. miR-9 targets AKT/GSK3β signaling pathway genes. miR-32 and miR-144 target PTEN expression. miR-29b regulates the proliferation and apoptosis of pulmonary artery smooth muscle cells. miR-155 regulates the adaptive immune response.	2012	[29]
		miRNA micro array	miR-424 and miR-365	miR-424 and miR-365 were up regulated in tuberculosis	miR-424 and miR-365 regulate cancer cell proliferation.	2011	[22]
		Small RNA sequencing	miR-23a-5p, miR-183-5p, miR-193a-5p, miR-941, miR-16-1-3p	miR-23a-5p, miR-183-5p, miR-193a-5p, and miR-941 were up regulated miR-16-1-3p was found to be down regulated	miR-23a-5p involves in the autophagy pathway. miR-183-5p promotes cell proliferation and migration. miR-193a-5p involves in proliferation and migration in cancer. miR-941 targets WNT16 to enhances osteoclastogenesis.	2020	[87]
5	Pleural Fluid	Small RNA sequencing	miR-3615, miR-320c, miR-205-5p, miR-218-5p, miR-135a-5p, miR-200a-5p, miR-320b, and miR-378i	miR-320c, miR-205-5p, miR-135a-5p, miR-200a-5p were up regulated and miR-3615, miR-205-5p, miR-320b, and miR-378i were down regulated in tuberculosis	miR-320c regulates inflammation. miR205-5p targets SMAD2 and regulates extracellular production. miR-135a-5p regulates adipogenesis. miR-200a-5p regulates myocardial necroptosis. miR-320c targets c-Myc and regulates cell proliferation.	2016	[36]
		Small RNA deep sequencing	miR-205-5p, miR-200c-3p, miR-429, miR-200b-3, miR-200a-3p, miR-203a-3p, miR-141-3p, miR-148a-3p, miR-451a, miR-150-5p	miR-205-5p, miR-200c-3p, miR-429, miR-200b-3, miR-200a-3p, miR-203a-3p, miR-141-3p, miR-148a-3p, miR-451a, miR-150-5p were up regulated in tuberculosis	miR-200 family members targets ZEB1/ZEB2 and regulates EMT. miR-429, miR-203a-3p, miR-451a, miR-150-5p regulate cancer cell proliferation. miR-141-3p regulates mesenchymal stem cells. miR-141a-3p regulates angiogenesis.	2017	[88]
6	Sputum	Exiqon miRCURYTM LNA arrays	miR-19b-2∗, miR-3179 and miR-147	miR-3179 and miR-147 were up regulated and miR-19b-2∗ was down regulated	miR-147 suppresses the expression of TNF-α and IL-6 and play as an anti-inflammatory miRNA.	2012	[49]
7	Urine	Real time PCR	miR-625-3p and miR-155	miR-625-3p and miR-155 were up regulated in tuberculosis	miR-625-3p regulates cancer cell proliferation. miR-155 regulates the adaptive immune response.	2018	[30]
8	EBC	miRNome array	miR-649, miR-1264, miR-2861, miR-574-5p, miR-453	miR-649, miR-1264, miR-2861, miR-574-5p, miR-453 were up regulated	miR-1264 targets DNA methyltransferase-1 and regulates its expression. miR-2281 and miR-574-5p regulate cancer cell proliferation.	2013	[89]

### 3.2 Common miRNAs identified in multiple clinical samples or multiple reports

By establishing differential expression, miRNAs can be used as potential biomarkers in different clinical samples from active tuberculosis patients. A biomarker indicates a normal biological process or a pathogenic process [23]. miRNAs are involved in many pathological processes and can be measured in various clinical samples. In the era of precision medicine, the identification and understanding of the function of specific miRNAs in clinical samples will play an important role. In recent studies, co-regulatory networks of miRNAs and their targeted transcription factor genes were analyzed in various clinical samples of tuberculosis patients [24,25]. Other studies have identified differential expression of miRNAs in patients with tuberculosis compared with healthy controls [26]. These studies may help us discover potential miRNAs as tuberculosis biomarkers. This review has screened published research articles that identified differentially expressed miRNAs in different clinical samples and shortlisted common miRNAs reported in more than one clinical sample in tuberculosis patients. The shortlisted common miRNAs are miR-155, miR-16, miR-200, Let-7, miR-486, miR-223, miR-99, miR-29, miR-21, miR-193, miR-365, miR-30, miR-20b, miR-146a, miR-31, miR-150 (Table 2).

**Table 2:** Differential expression of miRNAs in multiple clinical samples.

S. No	miRNAs	Remarks	Sample type	References
1	miR-155	Up regulated	Serum	[27]
			Plasma	[28]
			PBMCs	[29]
			Urine	[30]
2	miR-16	Up regulated	Serum	[20,32,90]
			Plasma	[34]
3	miR-200	Up regulated	PBMCs	[29]
			Pleural fluid	[36,88]
4	Let-7 family	Up regulated	Serum	[20,40]
			Plasma	[41]
5	miR-486	Up regulated	Serum	[43]
			Whole Blood	[44]
6	miR-223	Up regulated	Serum	[20]
			Plasma	[47]
7	miR-99	Up regulated	Serum	[27]
			Plasma	[47]
8	miR-29	Up regulated	Serum	[27,91]
			Whole Blood	[21]
			Plasma	[34,47]
9	miR-21	Up regulated	Serum	[27,90]
			Whole Blood	[21]
			Plasma	[28]
10	miR-193	Up regulated	Serum	[20]
			PBMCs	[29]
11	miR-365	Up regulated	Serum	[20]
			PBMCs	[22]
12	miR-30	Up regulated	Serum	[20]
			Whole Blood	[92]
			Plasma	[41]
13	miR-20	Up regulated	Serum	[58]
			Whole Blood	[44]
14	miR-146	Down regulated	Serum	[20]
			Whole Blood	[31,92]
			Plasma	[47]
15	miR-31	Down regulated	Whole Blood	[31]
			PBMCs	[60]
16	miR-150	Down regulated	Whole Blood	[21,31,92]

miR-155 is one of the extensively studied miRNAs found to be up-regulated in serum, plasma, PBMCs, and urine samples of tuberculosis patients [[27], [28], [29], [30]]. However, two reports found decreased levels of miR-155 in plasma and whole blood of tuberculosis patients [31,32]. miR-155 promotes the survival of tuberculosis-specific T cells and affects the adaptive immune response. Another report has suggested that serum miR-155 levels negatively regulated the TB-suppressing activity of natural killer cells. miR-16 was found to be elevated in both plasma and serum samples of tuberculosis patients [20,[32], [33], [34]]. According to a report, the levels of miR-16 decreased after anti-tuberculosis treatment [32]. However, miR-16 has been shown as a tumour suppressor miRNA for chronic lymphocyte leukaemia [35]. miR-200 was found to be elevated in pleural fluid collected from tuberculosis patients published in two different articles [29,36,37]. miR-200 family members could regulate mesenchymal to epithelial transition by regulating the expression of ZEB1/ZEB2 (zinc-finger- and homeobox-containing transcriptional regulator delta-crystalline enhancer-binding factor) and Smad-interacting protein 1 [38]. Another report suggests that miR-200 can inhibit adenocarcinoma [39]. Let-7 was found to be up-regulated both in serum and plasma samples of tuberculosis patients [20,40,41]. The let-7 family was involved in the pathogenesis by targeting various vital genes. Another report suggested that nuclear factor k beta (NF-kB) inhibits the let-7 family through activation of Lin28B, and the let7 family directly inhibited the expression of interleukin 6 (IL6) [42]. miR-486 was found to be up-regulated in whole blood and serum samples collected from tuberculosis patients [43,44]. miR-486 may regulate the nuclear factor of activated T-cells 5 (NFAT5) pathway genes [43]. miR-223 was found to be up-regulated in serum and plasma samples of tuberculosis patients compared with healthy controls [20,45]. miR-223 may regulate chemo-attractants including CXCL2, CCL3, and IL-6 in myeloid cells and control leukocyte chemotaxis [46]. miR-99b was elevated in serum, and whole blood samples of tuberculosis patients [27,47]. It was also found to be down-regulated in plasma after the anti-tuberculosis treatment. miR-29a was found to be up-regulated in serum and plasma samples of tuberculosis patients [21,27,[47], [48], [49]]. Interestingly, miR-29a was down-regulated in plasma samples after the treatment of tuberculosis. However, in another report, the levels of miR-29a were found down-regulated in whole blood samples [50]. miR-21 was found to be elevated in serum, whole blood and plasma samples collected from tuberculosis patients compared with healthy controls [21,27,28,51]. mir-21 targets TGF-β signalling pathway genes and regulates the expression [52]. It plays a crucial role in pulmonary fibrosis [53]. However, another report found the down-regulation of miR-21 in plasma samples [54]. miR-193 was up-regulated in PBMCs and serum samples in tuberculosis patients [20,29]. miR-193 plays an essential role in cancer cell proliferation [55]. miR-365 was up-regulated in serum and PBMCs collected from tuberculosis patients [20,22]. miR-365 regulates IL-6 production in macrophages, leading to the immune response in pulmonary tuberculosis [56]. miR-30 was found elevated in plasma, serum, and whole blood samples of tuberculosis patients [20,41,51]. During tuberculosis activation, miR-30 may suppress toll-like receptor/myeloid differentiation factor 88 (TLR/MyD88) activation and cytokine production [57]. miR-20b was found to be up-regulated in serum and whole blood samples collected from patients with tuberculosis [44,58]. However, published evidence suggested that miR-20b alleviates the inflammatory response in the tuberculosis mice model [59]. Another report showed the down-regulation of miR-20b in serum samples collected from tuberculosis patients [54]. miR-146a is also extensively studied miRNAs found to be down-regulated in plasma and whole blood samples collected from tuberculosis patients [20,31,47,51]. miR-31 was found to be down-regulated in whole blood and PBMCs samples collected from patients with tuberculosis [31,60]. According to another report, miR-31 was up-regulated in asthma patients [61]. miR-150 was down-regulated in the whole blood sample collected from patients with tuberculosis [21,31]. The report suggests that miR-150 plays an important role in B cell development [62]. However, the identified miRNAs are yet to be validated in all the clinical samples independently.

The discrepancies in the expression of miR-155, miR-29a, miR-21, and miR-20b in different clinical samples might be because of many reasons those influence the outcome of miRNA analysis [[63], [64], [65]]. These may include differences in the time of sample collection, comorbidities, and heterogeneity in the population studied. Other than this, the normalization methods used by different reports are different, and the effect of drug treatment on miRNA response is still unclear. Hence, a different set of subjects may give different expression patterns. Furthermore, different acquisition platforms and data-analysis pipelines used for miRNA quantification can influence the study outcome.

To overcome these issues, we reviewed articles showing the role of these miRNAs in the pathogenesis of tuberculosis. These miRNAs showed the potential role associated with the expression pattern reported in maximum articles. Hence, we precisely focused on the expression pattern of miRNAs reported in maximum articles.

### 3.3 miRNAs as potential biomarkers of tuberculosis

The human genome may encode more than two thousand functional miRNAs, which regulate most protein-coding genes. Interestingly, one miRNA can target multiple genes, and multiple miRNAs can regulate one gene [66]. According to various reports, miRNAs can regulate gene expression involved in the innate or adaptive immune pathways upon Mtb infection in the host [26]. Hence, knowing their association with any immune response, specific miRNAs can be used as a potential diagnostic or prognostic biomarker for tuberculosis. We reviewed the role of the miRNAs in the pathogenesis of tuberculosis. The shortlisted miRNAs including miR-155, miR-146a, miR-21 and miR-9, miR-125b, miR-26-5p, miR-132-3p can regulate innate immune cell activation ([26,54]). Interestingly, miR-155, miR-146a, and miR-21 have been identified in three or more clinical samples collected from tuberculosis patients. However, the rest of the miRNAs were identified in at least one clinical sample. miRNAs including miR-21-5p, miR-146a-5p, miR-20b-5p, miR-223-3p, miR-27b-3p, miR-99b-5p, miR-125-5p, miR-142-3p, miR-144, miR-27a are involved in the regulation of inflammation. MiR-21, miR-146, miR-20, miR-223, and miR-99 were identified in two or more clinical samples.

Recent studies have explored the role and regulation of other pathological processes (including autophagy and apoptosis) involved during TB infection. miRNAs including miR-155, miR-27a, miR-889, miR-106a, miR-125, miR-142-3p, miR-17, miR-144-3p, miR-20a, miR-23a-5p, miR-26a regulate the activation of autophagy to support the intracellular survival of Mtb. MiR-155 and miR-20 were identified in two or more clinical samples collected from tuberculosis patients. The rest of the miRNAs were identified in at least one clinical sample. Similarly, miRNAs including miR-155, miR-20a-5p, miR-21, Let-7e, miR-29a, miR-27b, miR-145 regulate apoptosis during Mtb infection. miR-27 and miR-145 were identified in at least one clinical sample, but the rest of the miRNAs were identified in two or more clinical samples. The summary of the targeted genes of the identified miRNAs and their roles in the pathogenesis of tuberculosis is mentioned in Table 3.

**Table 3:** Summary of the role of miRNAs in the pathogenesis of tuberculosis.

S. No	Function	miRNAs	Targeted Gene	References
1	Innate Immune response	miR-155	SHIP1 and TAB2	[93]
		miR-146a	IRAK-1/TRAF-6	[94]
		miR-21	PDCD4	[95]
		miR-9	NFkB1	[96]
		miR-125b	ERK1	[97]
		miR-26-5p	KLF4	[98]
		miR-132-3p	TLR	[99]
2	Regulation of inflammation	miR-21-5p	TLR4	[100]
		miR-146a-5p	TRAF-6	[101]
		miR-20b-5p	NLRP3	[102]
		miR-223-3p	NFIA	[103]
		miR-27b-3p	Bag2	[104]
		miR-99b-5p	TNF-α and TNFRSF-4	[105]
		miR-125-5p	TNF-α	[106]
		miR-142-3p	N-Wasp	[107]
		miR-144	INF-γ and TNF-α	[81]
		miR-27a	IRAK4	[108]
3	Autophagy	miR-155	Rheb	[109]
		miR-27a	Cacna2d3	[110]
		miR-889	TWEAK	[111]
		miR-106a	ULK1, ATG7, ATG16L1	[112]
		miR-125	DRAM2	[113]
		miR-142-3p	ATG16L1	[114]
		miR-17	ATG7	[115]
		miR-144-3p	ATG4a	[116]
		miR-20a	ATG7 and ATG16L1	[117]
		miR-23a-5p	TLR2/MyD88/NF-κB	[118]
		miR-26a	KLF4	[98]
4	Apoptosis	miR-155	FOXO3	[119]
		miR-20a-5p	JNK2	[120]
		miR-27b	Bag2	[104]
		miR-145	cIAP1	[121]
		Let-7e	Caspase 3	[122]
		miR-29a	Caspase 7	[123]
		miR-21	PI3/AKT/NFkB	[124]

## 4 Discussion

Accumulating evidence suggests that the differential expression of miRNAs may represent the differential pathogenesis of tuberculosis in different cell types. For example, in Mtb, infected macrophages derived from mouse bone marrow showed up-regulation of miR-155 and down-regulated in the infected macrophages derived from peripheral blood mononuclear cells (PBMCs) [15]. Upon Mtb infection, the expression of miR-155 get induced in murine derived macrophages and augment TLR signalling, thereby providing an environment for Mtb multiplication [67]. This promotes Mtb-specific T cells' survival and enables adaptive immune response. However, serum miR-155 levels show negative regulation of the TB-suppressing activity of NK cells [68]. miR-155 binds indirectly regulates TNF-α production [69]. Overexpression of let-7e, miR-29a, and miR-886-5p was found in Mtb infected macrophages derived from human monocyte [70]. Interestingly, target prediction analysis reveals that important apoptosis mediator caspases 3 and 7 are potential targets of let-7e and miR-29a. miRNAs profiling in Mtb infected human macrophages showed downregulation of miR-155, miR-146a, miR-145, miR-222, miR-27a, and miR-27b [71,72]. miR-145 is a pro-apoptotic miRNA found to be down-regulated, in line with a suppressed virulent Mtb strain to induce apoptosis [73]. miR-222, miR-27a, and miR-27b play crucial roles in controlling inflammatory response and lipid metabolism. Mtb was infected in human monocytes, and a miRNA expression profile was performed, where nine miRNAs including miR-30a, miR-30e, miR-155, miR-1275, miR-3665, miR-3178, miR-4484, miR-4668-5p, and miR-4497 were found to be differentially expressed [74]. miR-30a is activated by the TLR pathway and regulates inflammation and islet function by targeting interleukin one alpha (IL-1α) [57]. Another report showed that miR-30e inhibits apoptosis and promotes hepatocytes proliferation [75]. miR-1275 negatively regulates the expression of insulin growth factor (IGF) pathway associated genes and helps in the modulation of cell differentiation, proliferation, and apoptosis [76]. The IGF associated genes have been associated with LPS induced NFκB activation and pro-inflammatory cytokines production in human macrophages [77]. miR-3178 is known to target TNF receptor-associated factor 3 (TRAF 3) genes and ameliorates inflammation in gastric epithelial cells [78]. Bioinformatics analysis of miR-4484 suggests that the microRNA targets TGF-β and other Wnt/β-catenin signalling pathway-associated genes regulates apoptosis and is involved in collagen regulation related genes [79]. Another miRNA, miR-4668-5p, also targets TGF-β pathway-related genes and is involved in allergic diseases [80]. Other reports suggest that miR-144 expresses in T cells of active tuberculosis patients [81]. The up-regulation of miR-144 reduces T cell proliferation and inhibits the secretion of INF-γ and TNF-α.

Many research groups are identifying the detailed role of these differentially expressed miRNAs in the pathogenesis of tuberculosis. These studies may provide a deep understanding of tuberculosis pathogenesis and provide a good foundation for developing reliable biomarkers for the diagnosis.

However, the expression pattern of miRNAs ultimately depends on the study design. One miRNA may be differentially expressed in one study set, and the other studies may have used the same miRNA as a housekeeping gene. Similarly, one miRNA may be differentially expressed in certain disease conditions, and the same miRNA may also have shown differential expression in healthy controls in other studies.

The current article has focused on differentially expressed miRNAs in more than one clinical sample with the same expression pattern. However, a larger population size is required in different studies to establish miRNAs as potential biomarkers for tuberculosis. The diagnostic odds ratio of two oppositely expressed miRNAs may provide more accurate results as a diagnostic marker.

## 5 Conclusion

Tuberculosis remains one of the deadliest infectious diseases in the world. The causative agent, *Mycobacterium tuberculosis,* regulates the host defence system to survive and replicates through various pathways. In recent studies, the role of miRNAs in the innate and adaptive immune responses upon Mtb infection has been explored. miRNAs are involved in many pathways to alter immune responses by regulating various gene expressions in the host cell. However, there are some limitations in this regard. miRNAs are not entirely gene-specific or disease-specific. Apart from this, innate and adaptive immune response, autophagy and apoptosis are common phenomena in different inflammatory diseases. Same miRNAs can be involved in the pathogenesis of such inflammatory diseases, and it is challenging to separate tuberculosis from other inflammatory diseases based on the miRNAs signatures. Hence, multiple studies have focused on the signatures of shortlisted miRNAs and their expression associated with the pathogenesis of tuberculosis. With this aim, we have reviewed research articles published on differentially expressed miRNAs in clinical samples collected from tuberculosis patients and shortlisted miRNAs consistently shown in more than one clinical sample. This could help in identifying candidate miRNAs for tuberculosis detection.

In this review, we have found common miRNAs shown in various clinical samples in multiple studies. miRNA analysis was performed in the whole blood, serum, plasma, PBMCs, pleural fluid, sputum, urine, and EBC. Most miRNAs are detected in blood samples, which can easily be accessible from tuberculosis patients. Further, these miRNAs are also studied in the pathogenesis pathways of tuberculosis, such as innate and adaptive immune response, autophagy, and apoptosis. However, exploring the specific role of these miRNAs in pathogenesis is ongoing. Moreover, the identified miRNAs need to be validated in more extensive studies using *in vitro* and *in vivo* models. As the interest in this field increases, it is essential to identify other miRNAs biomarkers and a specific source of miRNAs in tuberculosis.

## Declaration of competing interest

The author has no conflicts of interest to declare.

## CRediT authorship contribution statement

**Bijay Pattnaik**: Writing the original draft, methodology, formal analysis, review & editing, **Niharika Patnaik**: Writing - review & editing, **Saurabh Mittal**: Supervision, Conceptualization, Methodology, Formal analysis, Resources, **Anant Mohan, Anurag Agrawal, Randeep Guleria**: Funding acquisition, Investigation, Project administration, Writing – original Writing - review & editing, **Karan Madan**: Supervision, Conceptualization, Methodology, Formal analysis, Resources.

## Funding

None.

## Financial disclosures

None of the authors have any financial disclosures.

## Declaration of interest statement

None.
